# A Call to Action for Gender Equity in Climate Leadership

**DOI:** 10.4269/ajtmh.22-0674

**Published:** 2023-05-01

**Authors:** Britt Wray, Erika M. Veidis, Elaine C. Flores, Allison A. Phillips, Ola Alani, Michele Barry

**Affiliations:** ^1^ Center for Innovation in Global Health, Stanford University, Palo Alto, California;; ^2^ Center on Climate Change & Planetary Health, London School of Hygiene and Tropical Medicine, London, United Kingdom

## Abstract

Climate action is not advancing quickly enough to prevent catastrophic harm. Understanding why might require looking at existing leadership structures and the inequitable gender representation therein. Critically examining dominant power structures could pave the way toward more comprehensive, innovative, and expedient environmental solutions—and we argue that elevating women’s climate leadership is key to safeguarding planetary health. Women have historically been left out of climate science and governance leadership. Women are disproportionately impacted by the health effects of climate change, particularly in Indigenous and low- and middle-income settings. Therefore, our call for women’s climate leadership is both an issue of justice and a matter of effectiveness, given evidence that inclusive leadership rooted in gender justice leads to more equitable outcomes. Here, we present evidence for why gender equity in climate leadership matters along with considerations for how to attain it across sectors and stakeholders.

## INTRODUCTION

Climate action is not advancing quickly enough. Understanding why might require looking at existing leadership structures. Women and those living in areas that suffer most from frontline effects of climate change, particularly in low- and middle-income countries (LMICs), are often excluded from power-holding positions in climate governance yet have contributed the least to environmental degradation.^1^^–^^6^ Intersectional approaches that recognize the economic, geographic, cultural, racial, and other often intersecting factors feeding the inequitable impacts of climate change are critical foundations for finding and employing equitable solutions.^7^ Equitable solutions require perspectives outside of dominant systems, and engaging the voices, values, and ideas of underrepresented groups is critical to accelerating urgently needed, practical, and life-sustaining climate and environmental action.

In Audre Lorde’s words, “the master’s tools will never dismantle the master’s house.”^8^ In this piece, we examine this adage through the lens of gender—and argue that, in the face of accelerating environmental change, humanity cannot afford to leave women out of climate leadership. Gender equity in climate leadership across axes of governance and policy, business, academia, the nonprofit sector, and implementation groups may be a critical lifeline to securing a sustainable future in which humanity can thrive.†

## EMPOWER WOMEN FOR MORE EFFECTIVE CLIMATE SOLUTIONS

Women’s leadership in government corresponds with greater environmental protections,^10^ more stringent climate policies,^11^ and the ratification of environmental treaties^12^—as seen, for example, in increased favorable environmental outcomes in the European Parliament and U.S. Congress.^13^^,^^14^ Moreover, the environmental and climate policies that do get passed when women are in leadership positions are likely to be more effective and promote equality.^15^

This holds at scale—and also at the grassroots level. For example a study of Community Forestry Institutions (CFIs) in India and Nepal that analyzed data across 94 villages found that women’s presence in the Executive Committees of CFIs positively impacts forest conservation and women’s empowerment, justice, and welfare.^16^ Another study looking at the impact of enforcing gender quotas in forest management community groups in Indonesia, Peru, and Tanzania found that groups in which women comprised an equal number or majority of members conserved more trees and shared payments more equally among members.^15^

Those who control resources usually have the power to monopolize conversations and perpetuate existing narratives, but if these trends can be disrupted, so too might the current stagnation in effective climate action and other needed pro-environmental policies.^17^ By advancing women in climate leadership and policy, we argue that this generation and those that follow will stand a better chance of accelerating climate solutions.

## HOW WOMEN ARE LEFT OUT OF CLIMATE LEADERSHIP

The barricades against women’s climate leadership are as old as the field of climate science itself. Even women responsible for scientific breakthroughs foundational to climate science have been left out of the dialogue and predominantly white and male-dominated existing leadership structures.

History books, for example, do not teach that it was a woman, Eunice Foote, who first discovered that carbon dioxide is what we now call a “greenhouse gas,” and instead credit male scientist John Tyndall for that breakthrough.^18^ This example illustrates the Matilda effect^19^—a bias against acknowledging women for their scientific accomplishments in favor of men with more opportunity for visibility and impact. Today, we see bias against women’s leadership continuing at larger scales in global climate policy and scientific decision-making.

Although women’s representation in the United Nations’ (UN’s) Intergovernmental Panel on Climate Change (IPCC), for instance, has grown from 8% in 1990 to 33% in 2021, a recent survey examining gender balance in the IPCC found limited female representation and engagement.^20^ Despite women constituting 30% of researchers worldwide, there were fewer women in senior research positions contributing to it.^21^ In addition, women reported unequal opportunities to be nominated, speak, shape content, or lead IPCC chapters. Male scientists were perceived to dominate discussions and writing, and several instances of women being uncredited, ignored, or even harassed were reported. Furthermore, of the top 1,000 influential climate scientists measured by publication records and compiled by Reuters, only 12.3% are women, and of the top 20 climate scientists, only one is a woman, pointing out a significant gender gap in climate science.^22^

Gender inequality is a destructive force that undermines women’s rights and abilities to lead in entrepreneurship, governance, research, and economic participation. Early influences that shape these outcomes are embedded within childhood education and the provision of other privileges and resources. For instance, well-documented disparities exist in how girls and boys are educated, particularly in science, technology, engineering and math fields.^23^^–^^25^ This imbalance leads to a reduced overall comparative pool of credible women scientists and professionals, affecting women’s ability to hold power over multiple matters given current economic and governance paradigms. The gender gap is troubling and stark across positions of power inside the institutions most capable of swiftly moving the needle on climate action, particularly in national governments that are sent to the UN climate negotiations. This is symptomatic of a broader global political leadership problem, in which there is a dearth of women at the top.^26^

Efforts to remedy this trend in climate governance have not yet succeeded in making systemic change. Several attempts at the UN level have sought to advance women’s leadership on climate, including the Glasgow Women’s Leadership Statement on Gender Equality and Climate Change launched at COP26 in a joint effort by the Scottish Government and UN Women (COP, or Conference of the Parties, is an international climate summit brought together by the United Nations Framework Convention on Climate Change [UNFCCC])^27^; the UN Secretary General’s initiative on Gender and Climate Change launched at the Global Climate Action Summit 2019^28^; the Feminist Action for Climate Justice Action coalition under the Generation Equality Forum^29^; and the UN Women’s “Feminist Plan for Sustainability and Social Justice” report,^30^ as well as the Gender Action Plan agreed on at COP25 to advance the rights and interests of women and girls in the UNFCCC process and support gender-responsive climate policy and programming.^31^ However, these initiatives risk being mere symbolic gestures, as the UN climate negotiations remain dominated overwhelmingly by men.

At the 2021 COP26, only 33% of all formal roles were filled by female government delegates, as was the case in 2020 and 2019, demonstrating the lack of progress being made in female representation.^32^ Women from the most climate-vulnerable countries were largely absent at decision-making levels. This occurred despite widespread acknowledgment at the negotiations of the disproportionate vulnerability that women face from climate change, especially in LMICs and Indigenous communities.^27^ Ahead of the gathering, 400 influential women publicly challenged the COP26 United Kingdom hosting team (as coordinated by the global campaign and advocacy group She Changes Climate) and demanded/by demanding that the predominantly male COP26 steering team shift to 50:50 gender representation,^33^ a plea that was left unmet. The following year, COP27 in Egypt was led by another male president, UN Climate Change mentioned that women led only 20% of national delegations,^34^ and according to She Changes Climate, only seven of the 110 world leaders at COP27 were women.^35^ Since the first COP in 1995, only four women have been appointed as COP presidents.^35^ The United Arab Emirates has named a male oil company chief to lead the COP28 climate negotiations next year.^36^

## WOMEN AND GIRLS FACE ELEVATED HARM IN A WARMING WORLD

Women bear the brunt of harm and health risks from climate hazards,^37^ especially in emerging economies,^38^ and have been historically deprived of agency and voice. In addition to biological susceptibilities,^39^^,^^40^ especially during pregnancy, climate change disproportionately harms women as a result of traditional roles and preexisting structural and societal inequities that impair women’s influence and status^2^^,^^41^^,^^42^ (Table 1).

**Table 1:** Disproportionate impacts of climate change on women

Pathway	Gender specific outcomes
Disaster-related mortality and resilience	Women are more exposed to climate disasters and may suffer disproportionate mortality rates from these events.^43^^–^^45^
	Particularly in low- and middle-income countries, women often have less access to financial resources (savings, loans, credits) that are protective in emergencies and influence both disaster preparedness and recovery.^46^^–^^48^
Infectious disease	Climate change is increasing the emergence and spread of infectious diseases.^49^ This disproportionately affects women and girls in lower-income settings owing to their increased contact with vectors through traditional water collection.^38^^,^^50^^–^^52^
	The growth of climate migration as a global phenomenon will increase the risk of disease spread by gender-based violence, with subsequent sexually transmitted diseases occurring during displacement.^51^,^46^^–^^48^
Food insecurity	An estimated 60% of chronically hungry people are women and girls,^53^ a population that also experiences higher rates of anemia and malnutrition.^44^
	Women often occupy unfavorable positions in household food hierarchies in lower-income settings, exacerbating their hunger.^54^
	Climate change can both decrease access to food and diminish the nutritional quality of food,^55^ magnifying these risks for women.
	Agricultural impacts of extreme weather events have been found to be worse in areas where land shares are owned by women, potentially because of less investment in agricultural technologies, impacting women’s welfare.^56^
Reproductive health	Climate and environmental hazards such as pollutants affect women’s reproductive health and fertility^57^^,^^58^ and impact their ability to access reproductive and maternal health services.^44^
	Pregnancy-related outcomes can be affected by infectious disease, nutrition risks, and extreme heat,^44^ all of which are exacerbated by climate change.
	Heat stress, especially prevalent in lower-income regions, can increase the occurrence of preterm births, low birth weight, developmental malformations, and stillbirths following maternal exposure to extreme heat.^59^
Air quality	Poor air quality made worse by climate change–related wildfire smoke, drought-related dust, and ground-level ozone disproportionately impacts women given their higher rates of anemia and higher propensity to have particulate matter deposited in the lung tissue.^44^
	Cooking indoors with solid biomass endangers the respiratory health of women and girls worldwide—who traditionally occupy cooking roles—by increasing their exposure to air pollution.^60^^–^^62^
Heat	The relationship between heatwaves and human morbidity and mortality is well established.^63^ This is especially the case in lower-income countries, which bear a greater burden of extreme heat events and drought.^64^
	Across lower-income regions, women and girls face increased health risks from heat because of their lower socioeconomic status and traditional gender roles, such as carrying wood for cooking.^54^
Mental health	Women experience greater climate change–related mental health concerns, such as anxiety and depression.^65^
	Women’s traditional roles as caregivers—and in some settings, closer day-to-day interactions with the land—could place them at higher risk for mental health impacts during and after environmental events.^66^^,^^67^
Violence	Gender-based violence—including sexual assault, domestic abuse,^44^^,^^68^ and intimate partner violence^69^—has been found to increase during and after extreme weather events and is often related to economic instability, food insecurity, mental stress, damaged infrastructure, increased exposure to men, and patriarchal norms.^70^^,^^71^
Displacement	According to the United Nations, 80% of people displaced by climate change are women.^72^ Displacement is typically linked to the underserved and impoverished, which itself is significantly impacted by gender.^73^
	The increase in climate disasters and displacements, including the loss of property, livelihoods, and other indirect and long-term effects, is known to have a gender-differentiated impact.^74^
	In some settings, natural disasters have been found to have stronger and longer-lasting consequences for women’s economic status.^75^

## WHY WOMEN’S LEADERSHIP MATTERS: A CALL TO ACTION

We call for more women in global climate leadership and policy. More specifically, we call for equitable representation, meaning at least 50% female representation from women leaders with environmental expertise and lived experience, in governments in high-, middle- and low-income countries and in multilateral climate organizations, including the IPCC and all upcoming UN climate negotiations at COP. Women should also be elevated in other spheres of climate action—including the private sector, academia, local institutions, and community organizations, all of which are central to comprehensive adaptation and mitigation.

We recognize that women’s climate leadership, especially from disadvantaged settings, is obstructed by multiplicative structural factors,^20^ including cultural norms, childcare obligations, inadequate financial support, and limited access to education. Ensuring gender equity in leadership means enacting structural changes to address these disparities in ways that are accountable to populations. Nations must invest in equitable access to childhood education and other resources that cultivate mastery of leadership assets to help close the gender gap in climate leadership. Paying attention to this confluence of issues reveals how acting more effectively on climate change may simultaneously require addressing women’s education, economic empowerment, and access to reproductive healthcare, childcare, and other support services.

Although the work of describing how we may disassemble structural barriers is beyond the scope of this paper, we summarize our call to action as the need to see equal representation of female climate leaders appear in^76^ 1) governments in high-income countries (i.e., the governments that must provide their fair share in climate finance alongside implementing ambitious emission reduction targets, as they are responsible for more than 79% of historic carbon emissions)^77^; 2) governments, local institutions, and communities in LMICs (i.e., bodies that must enact policies and actions that are relevant to climate adaptation and disaster risk reduction); 3) multilateral organizations (i.e., the institutions that must implement pro-poor climate-relevant policies), including the IPCC and upcoming UN climate negotiations at COP, paying particular attention to making sure that women from LMICs and climate-vulnerable populations are represented; and 4) climate research and academia, ensuring that the data and findings that often form the foundation for many decisions, new innovations, and policies represent a broad perspective.

In conclusion, tackling the enormous challenge of climate change requires listening to the world’s most vulnerable populations, recognizing how innovative and transformative thinking requires drawing from the voices most impacted by climate challenges yet often left out of current power-holding positions, and designing comprehensive approaches to elevate equitable leadership. In this case, our focal population is women—but the argument is similar for Indigenous populations, communities of color, lower-income communities, and others who have been marginalized by cultural, political, and economic norms and are acutely impacted by climate disruption and other environmental challenges.

Diversifying climate leadership by empowering women is a critical missing catalyst to launch us into a new era of desperately needed solutions, incisive decision-making, and urgent action for a healthier planet that supports the thriving of all its people.
